# Geometry of anchoring miniscrew in the lateral palate that support a tissue bone borne maxillary expander affects neighboring root damage

**DOI:** 10.1038/s41598-021-99442-2

**Published:** 2021-10-06

**Authors:** Song Hee Oh, Sae Rom Lee, Jin-Young Choi, Hyo-Won Ahn, Seong-Hun Kim, Gerald Nelson

**Affiliations:** 1 Department of Oral and Maxillofacial Radiology, Graduate School, Kyung Hee University, Seoul, Korea; 2 Department of Orthodontics, Graduate School, Kyung Hee University, Seoul, Korea; 3 Division of Orthodontics, Department of Orofacial Science, University of California San Francisco, San Francisco, CA USA

**Keywords:** Anatomy, Oral anatomy

## Abstract

Anchoring miniscrews used for a tissue bone borne maxillary expander (C-expander) can fail if they contact tooth roots or perforate the maxillary sinus. Cone beam computed tomography images were reviewed retrospectively to evaluate the geometric factors of miniscrew placement in the palate that contribute to root proximity (RP) and sinus perforation (SP), and to investigate the differences of miniscrew placement depth (PD) and placement angle (PA) among the groups in each variable from 340 anchoring miniscrews on 70 patients whose C-expanders showed sufficient stability after palatal expansion for orthodontic treatment. Two types of miniscrews were used: a self-tapping miniscrew with 1.8 mm-in-diameter, and a self-drilling miniscrew with 1.6 mm-in-diameter. While the self-tapping larger diameter miniscrew influenced root proximity significantly, the screw location and PD affected the rate of sinus perforation. PA was significantly different between the right and left sides of the palate. The results of this study confirmed that root proximity and sinus perforation of anchoring miniscrews in a tissue bone borne palatal expander occurred due to certain risk factors, even when the palates were expanded successfully. Knowledge of these factors can help the clinician place miniscrews with less risk of root proximity or sinus perforation.

## Introduction

Maxillary transverse deficiency is a major problem in orthodontics, and may be accompanied by unilateral or bilateral posterior cross-bite or arch length discrepancy^1, 2^. This malocclusion is very unlikely to correct spontaneously, and can adversely affect maxillofacial growth as well as the developing permanent dentition^1–3^. For treatment, the narrow palate is expanded transversely. Rapid maxillary expansion (RME) is one of the treatment modalities. The most common design of a device for RME has been a tooth anchored expander with or without an acrylic plate^4, 5^. Use of the conventional RME is accompanied by limited skeletal movement, dento-alveolar tipping, root resorption, periodontal dehiscence, and alveolar bone loss^4–7^. To more effectively separate the basal bone and avoid negative effects for adults, surgically assisted rapid palatal expansion (SARPE) has been recommended^8–11^. However, this requires invasive surgery, patient inconvenience and extra cost. In order to avoid the disadvantages of conventional RME with SARPE, clinicians have used temporary skeletal anchorage devices (TSADs) to support the expanders^12–15^. Two examples of skeletal maxillary expanders are the miniscrew-assisted rapid palatal expander (MARPE), a tooth bone-borne expander, and the Biocreative expander (C-expander)^16^, a tissue/bone-borne expander^14, 17, 18^. MARPE is a modified version of the conventional RPE device, using several miniscrews to expand the basal bone and to maintain the bone separation during expansion. The therapeutic effect of this device is a combination of skeletal and dentoalveolar expansion^17^. The C-expander is supported with miniscrews in the lateral palatal wall, which are placed between the canines and the first premolars, between the first and second premolars, and between the second premolar and first molars, providing a force distribution similar to the hyrax appliance, but without tooth contact. The acrylic plate distributes the palatal stress, decreasing concentrated stress on the implants^14, 19–21^. Parallel dentoalveolar expansion occurrs with minimal buccal dental tipping. One study of the tooth/bone-borne expander shows much less dental tipping than the conventional RME^20^. Another study of the skeletal and dental changes with a bone-borne expander indicated effective palatal widening with negligible dental effects^22^.

When miniscrews are placed in the inter-radicular space of the palatal slope, one must consider risks to the adjacent roots. Root contact can lead to TSAD loosening^23, 24^. Min et al. studied the relationship between root proximity and the success rate of miniscrews using cone-beam computed tomography (CBCT) and concluded that root proximity limits the success rate^25^. Although another study suggested that root proximity was not the main risk factor for miniscrew failure^26^, TSAD/root contact can damage the root^27^. There are few studies about potential risk factors of palatal miniscrews to damage the dental roots and sinus walls.

Therefore, this study aimed to evaluate whether various parameters, such as skeletal classification, miniscrew geometry, placement side, or screw location, affect the risk of root proximity and sinus perforation, and to investigate differences of miniscrew placement depth or angle among the groups in each variable using three-dimensional cone-beam computed tomography (CBCT) imaging.

## Results

A total of 346 miniscrews were included in this study. Six out of 346 screws migrated outside of the alveolar bone during expansion by the pushing force from the expander, and the overall success rate was 98.3%. Of 6 deviated miniscrews, 4 miniscrews were type A, and the remaining 2 miniscrews were type B. Statistical analysis was performed on a total of 340 miniscrews. Out of 340, 58 miniscrews (17.1%) had root contact and 46 miniscrews (13.5%) had maxillary sinus perforation. The average of PD was 2.8 mm, and the average of PA was 35.3° (Table 1).

**Table 1:** Sample distribution according to variables.

Variables	*N* (%)
Total number of miniscrews	340
**Skeletal classification**	
I	99
II	82
III	159
**Screw type**	
A	120
B	220
**Placement side**	
L	170
R	170
**Screw location**	
#3	94 (27.6%)
#4	82 (24.1%)
#5	90 (26.5%)
#6	74 (21.8%)
**Root proximity**	
No contact	282 (82.9%)
Contact	58 (17.1%)
**Sinus perforation**	
No	294 (86.5%)
Yes	46 (13.5%)
Placement depth	*Mean* (mm)
	2.8
Placement angle	*Mean* (°)
	35.3

L left, R right; #3 Canine, #4 1st premolar, #5 2nd premolar, #6 1st molar.

The results of root proximity and sinus perforation according to each variable are presented in Table 2. There was a significant difference in root proximity according to the screw type. In type A miniscrews, root contact was observed in 35 (29.2%) out of 120, whereas type B miniscrews showed 23 (10.5%) root contacts out of 220 miniscsrews. In the case of sinus perforation, location was the correlating factor. The probability of sinus perforation was statistically significantly lower when the miniscrews were implanted in the anterior palatal slope than when they were placed in the posterior palatal slope.

**Table 2:** Root proximity and sinus perforation according to each variable.

Variable	Root_proximity		*p*-value	Sinus_perforation		*p*-value
	No contact	Contact		No	Yes	
**Skeletal classification**						
I	83	16	0.8828	91	8	0.1956
II	66	16		72	10	
III	133	26		131	28	
**Screw type**						
A	85	35	0.0009*	104	16	0.9714
B	197	23		190	30	
**Placement side**						
L	142	28	0.7537	149	21	0.5394
R	140	30		145	25	
**Screw location**						
#3	72	22	0.818	96	1	0.0007*
#4	67	15		74	6	
#5	83	7		70	22	
#6	60	14		54	17	

L left, R, right; #3 Canine, #4 1st premolar, #5 2nd premolar, #6 1st molar.

*p < 0.05.

The mean values and standard deviations of PD and PA, according to each variable, are presented in Table 3. The PD value showed a significant difference according to the location of the screw. The deepest placement depth (3.23 mm) was in the first molar area. Sinus perforation was statistically related to the placement depth. In the cases with sinus perforation, the PD was 3.14 mm, significantly higher than the PD (2.80 mm) in the absence of sinus perforation. PA showed smaller amount on the left side than the right side. There was a positive correlation between PD and PA (Table 4).

**Table 3:** The mean values and standard deviations of placement depth and placement angle according to each variable.

Variable	Placement_depth (mm)		*p*-value	Placement_angle (°)		*p*-value
	Mean	SD		Mean	SD	
**Skeletal classification**						
I	2.91	0.83	0.8816	36.50	13.68	0.6592
II	2.80	0.84		35.76	13.51	
III	2.83	0.90		34.37	12.74	
**Screw type**						
A	2.91	0.95	0.5640	37.94	15.25	0.1453
B	2.81	0.81		33.90	11.73	
**Placement side**						
L	2.82	0.91	0.5109	36.48	13.26	0.0217*
R	2.87	0.82		34.17	13.08	
**Screw location**						
#3	2.83	0.75	0.0005*	34.10	12.71	0.3021
#4	2.63	0.74		36.67	13.30	
#5	2.74	0.84		34.61	14.22	
#6	3.23	1.05		36.42	12.40	
**Root proximity**						
No contact	2.81	0.86	0.2684	34.23	12.99	0.1941
Contact	2.98	0.91		40.64	13.02	
**Sinus perforation**						
No	2.80	0.83	0.0197*	35.35	13.41	0.8846
Yes	3.14	1.04		35.19	11.93	

L left, R, right; #3 Canine, #4 1st premolar, #5 2nd premolar, #6 1st molar.

*p < 0.05.

**Table 4:** Correlation of placement angle and placement depth.

Variable	Placement_Angle					
	Spearman's correlation	*p*-value	Mixed model (dep = Placement_Angle)			
			B estimate	CI		*p*-value
Placement_depth	0.19	0.0003	3.57	2.14	5.00	< 0.0001*

*p < 0.05.

## Discussion

RME is commonly used for orthodontic treatment of patients with transverse maxillary deficiency, dental crowding, and/or mandibular functional shift. Skeletally transverse expansion separates the maxilla by opening the midpalatal suture^28^. In order to achieve successful midpalatal suture opening, a variety of bone-borne type expanders are available. The tissue-and-bone-borne maxillary expander (C-expander, no-tooth contact) is associated with less buccal bone loss. Moon et al. evaluated molar inclination and surrounding alveolar bone change when comparing the MARPE and C-expander^29^. Although skeletal expansion was similar in the MARPE and C-expander groups, the degree of dental expansion in the C-expander group was about half of that in the MARPE group. Dental expansion in the MARPE group was associated with a decrease in buccal alveolar bone height and thickness. The anchoring miniscrews of C-expander are placed in the inter-radicular space of the palatal slope, with a potential risk to the adjacent root damages^21, 29, 30^. This is why we wanted to analyze the specific features of placement of the TSADs placed in the inter-radicular space.

For the C-expander, we used two types of TSADs (Type A: self-tapping miniscrew with 1.8 mm in diameter, Type B: self-drilling miniscrew with 1.6 mm in diameter). Considering the palatal inter-radicular space, there is no interdental site greater than 2 mm except for the area between the second premolar and the first molar, according to a previous CBCT study^30^. At interdental sites from the canine to the second premolar, there is a low possibility of the miniscrew tip approaching the narrowest inter-radicular area at interdental sites, since the hard and soft tissue thickness is > 8 mm. In the canine, premolar, and molar regions, the environment of the soft-tissue and palatal bone thickness, and the inter-radicular space provide favorable bone support for the miniscrew. In this study, out of 340 miniscrews, 58 had root contact (17.1%). The incidence of root proximity differed significantly according to screw type and screw location. Type A miniscrews with a larger diameter increased the probability of root contact. Root proximity is considered to have a strong correlation with miniscrew failure, as shown in the animal study of Chen et al.^31^. According to Kuroda et al.^24^, root proximity was a major risk factor for miniscrew failure. Shinohara et al.^32^ reported that there was a statistically significant difference in the failure rate of miniscrew with and without root contact (*p* < 0.001). However, in our study, approximately 80% of miniscrews with root contact survived, and most of the 1.6 mm diameter miniscrews with root contact survived. Min et al.^25^ studied the relationship between the success rate and root proximity of slender miniscrews with a diameter of 1.2 to 1.3 mm. They reported that 16 of 172 (9.3%) had root contact and 11 failed (failure rate, 68.8%). This low rate of root contact but high failure rate might be related to the small diameter of miniscrew. Apparently, root proximity and screw diameter were relevant to stability. In the Kim et al.’s CBCT study, root contact of a miniscrew with root proximity only on one side showed high stability^26^. All the miniscrews with root contact in this study showed only one side contact, which may explain the success of the miniscrews.

Maxillary sinus penetration is considered a potential risk factor in miniscrew retention^33^. In this study, 46 of the 340 miniscrews (13.5%) perforated the maxillary sinus. However, a perforation less than 1.5 mm does not affect the stability of miniscrew. In addition, orthodontic treatment is generally not hindered by sinus perforation, since the perforation of the maxillary sinus can heal spontaneously^34^. Pneumatization of maxillary sinuses is more pronounced in the posterior teeth area than anterior area^35^. Since the anterior region has narrower inter-radicular spaces and thicker hard tissue than the posterior region, sinus perforation is less likely^30^. In addition, as the width of the arch becomes narrower anteriorly, deeper miniscrew placement is inadvisable^36^.

The average PA of the miniscrews was 36.48° on the left side and 34.17° on the right side. Kim et al.^26^ measured the PA of a miniscrew placed in the maxillary buccal alveolar bone and found that there was a large difference in the vertical angle between the right and left sides in the front. This difference might be a clinician factor due to the difference in visual confirmation of the angulation. PA differences of the palatal miniscrews between right and left side showed opposite results to the maxillary buccal miniscrews results. In this study, the average value of the PD was 2.8 mm. PD is defined as the length of the miniscrew that penetrates the bone, excluding the soft tissue thickness. Palatal soft tissue is thicker than buccal soft tissue. According to Lee et al.^30^, the average palatal soft tissue thickness was 3.56 mm, and suggested that the average PD of 2.8 mm would be a thickness that does not affect stable maintenance. PD also showed statistical differences according to the screw location in this study. Deeper bone penetration in the first molar area was likely related to thinner soft tissue. According to previous research, the palatal soft tissue at the first molar was the thinnest^30^. We also found a positive correlation between PD and PA. A larger PA means a more perpendicular placement of the miniscrew to the bone surface, with the PD increased accordingly. Skeletal classification and screw type were not significantly related to PD. Because patients who used the C-expander were patients with maxillary constriction regardless of the type of malocclusion, the placement condition was almost same among the skeletal classification I, II, and III. Type A and B screws had similar length. Although the most obvious difference between the screw types A and B was the diameter, their lengths were similar to each other, having no significant effect on the PD. For mechanical maintenance of the C-expander, PD was a variable that was considered more than root proximity. Initial stability was obtained only when it was placed beyond a certain depth with a certain degree to the bone surface.

The overall success rate for TSAD placement was 98.3%. The sample size and the number of miniscrews placed were sufficient to validate this success rate. Most of the miniscrews withstood static lateral forces. A high success rate was likely due to the broad force distribution among 4 to 6 screws and the osseointegration potential of the surface treated type A miniscrews^26, 29^. Four of the deviated six miniscrews were type A, likely due to the influence of micromotion of adjacent soft tissues during the healing period or root proximity due to the larger diameter. We do not claim to objectively evaluate placement technique, as only one experienced orthodontist placed all the miniscrews used in this study. Executing the exact plan for placement position was limited since supportive tools such as a screw guide were not used.

A limitation that is common to all studies using CBCT for measurements is the spatial resolution of CBCT. Important factors influencing spatial resolution are the partial volume effect, artifact, and scattering radiation^37^. The partial volume effect occurs when two structures with different densities are included in a particular voxel. The density of this voxel is assigned to the average density of the two tissues. These characteristics of CT have limitations in clear visualization of delicate anatomical structures such as alveolar bone plates^38^. For quantitative assessments, the partial volume effect may cause over- or under-estimate measurements. Blooming is an artifact commonly observed in CBCT images. It has reported that the artifact depends on the density of materials, such as root canal sealers, implants, metal crowns, and other restoration materials present in the volume scanned^39^. Such artifact results in an increased perception of the volume of the metal objects, which may lead to false positive assessments of root proximity or sinus perforation. Scatter radiation in CBCT increases as the size of the FOV increases. The larger the FOV is, the lower the resolution of the image. This can make it very difficult to detect thin bone less than 1 mm. The appropriate way to prevent poor resolution is to use the smallest FOV that encompasses the region of interest^40^.

When it comes to the radiation issues, there have been controversies about performing CBCT routinely for orthodontic patients, especially in young patients. As part of the routine treatment protocol for orthodontic treatment, CBCT used in this study was taken only in patients with a special purpose of evaluating the treatment outcome and prognosis after maxillary expansion, in cases which had definite benefits. If the benefits were higher than the expected risks, taking required radiographs could be considered to be ethically sound.

In this study, the CBCT was not taken immediately after miniscrew placement in consideration of the radiation dose burden. That was the reason we could not study the displacement tendency or risk factors related to the characteristics of expansion forces. Further stability studies could look at the three-dimensional PD specifics, the possibility of adjusting the expansion vector according to the placement position, the relationship between the amount of suture opening/expander activation efficiency, sinus/root perforation by the anchoring miniscrews, and the placement method of miniscrews.

## Conclusion

The results of this study confirmed that the root proximity or sinus perforation of anchoring miniscrews in a tissue/bone-borne palatal expander was connected to certain risk factors, even in the cases with successful expansion. The risk of root proximity was higher with the self-tapping miniscrew with a larger diameter (Type A) than the self-drilling miniscrew with a smaller diameter (Type B). The probability for perforation of the maxillary sinus was significantly higher toward the posterior palatal slope since there were larger sinus volumes. Miniscrews at the first molar area were placed in a deeper depth, likely due to a thinner soft tissue covering. Knowledge of these factors can help the clinician place miniscrews with less risk of root proximity or sinus perforation.

## Methods

### Subjects

It was a retrospective study on consecutively treated patients. The subjects were 70 patients (27 men, 43 women; aged 13–38 years; mean age 20.2 years) who had maxillary expansion treatment using a tissue and bone-borne type skeletal maxillary expander in the Department of Orthodontics at the Kyung Hee University Dental Hospital from 2011 to 2020.

CBCT radiographs were taken for orthodontic treatment evaluation only. All the experimental protocol with informed consent from all participants and the legally authorized representatives/parents/guardian/next of kin (in case of minors) for study participation was approved by the institutional review board of Kyung Hee University Dental Hospital (IRB No. KH-DT20012). The authors confirm that all methods were carried out in accordance with relevant guidelines and regulations.

The inclusion criteria were as follows: CBCT images acquired 3 months after expansion, a transverse maxillary deficiency, permanent dentition, and over 7 mm of expander activation without any surgical treatment during the expansion period. Exclusion criteria included systemic diseases, craniofacial anomalies, a failure of midpalatal suture separation, or deformation of the expansion device.

### Description of the TSADs and the activation protocol

**Figure 1:**
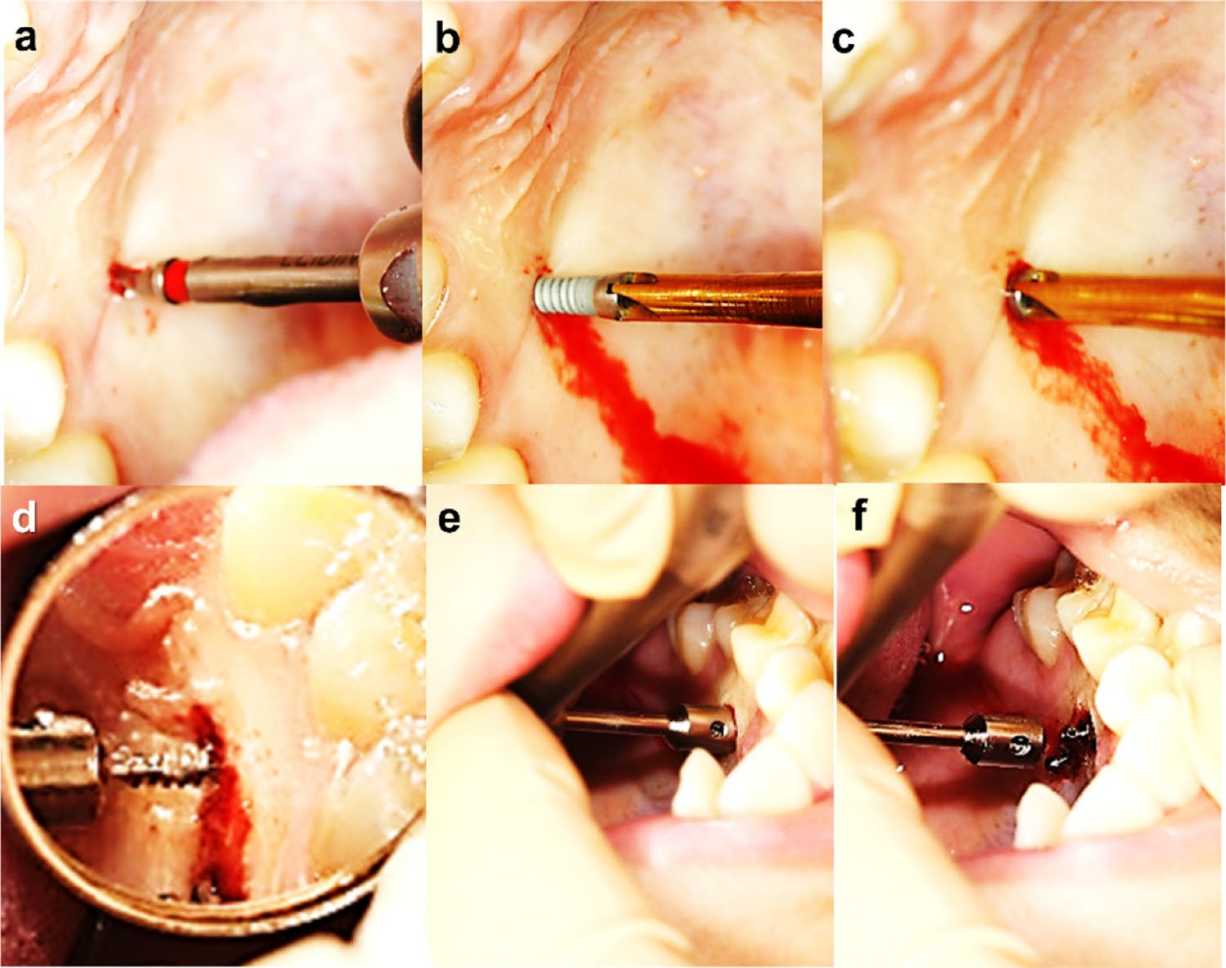
Placement procedure of anchoring miniscrew. (**a**) Predrilling with 1.5 mm in diameter guide drill for the self-tapping C-implant. (**b**, **c**) 1.8 mm in diameter 9.5 mm in length screw part (8 mm of miniscrew under the mucosa) is placed using a manual driver, (**d**–**f**) the 1.6 mm in diameter 8 mm in length self-drilling miniscrews is placed without predrilling.

The tissue bone-borne type skeletal maxillary expander (C-expander) is composed of expansion screws with an acrylic resin base supported by anchoring TSADs (Forestadent, Bernard Förster GmbH, Pforzheim, Germany). There were two types of TSADs used at this study, of which type A was sand-blasted with a large-grit and acid etched (SLA) surface treated self-tapping miniscrew (C-implant; Cimplant Co, Seoul, Korea), with a diameter of 1.8 mm and a length of 9.5 mm (Screw part for bone penetration is 8.0 mm in length). Pilot drilling is needed for the self-tapping miniscrew insertion (Fig. 1a–c). Type B is a machined self-drilling miniscrew (Bio-Action screws, Jin Biomed co., Bucheon, Korea), with a diameter of 1.6 mm and a length of 8.0 mm. This does not need any pre-drilling during miniscrew insertions (Fig. 1d–f). The number of patients who used C-expander with type A screw was 32 and those who used type B were 38.

**Figure 2:**
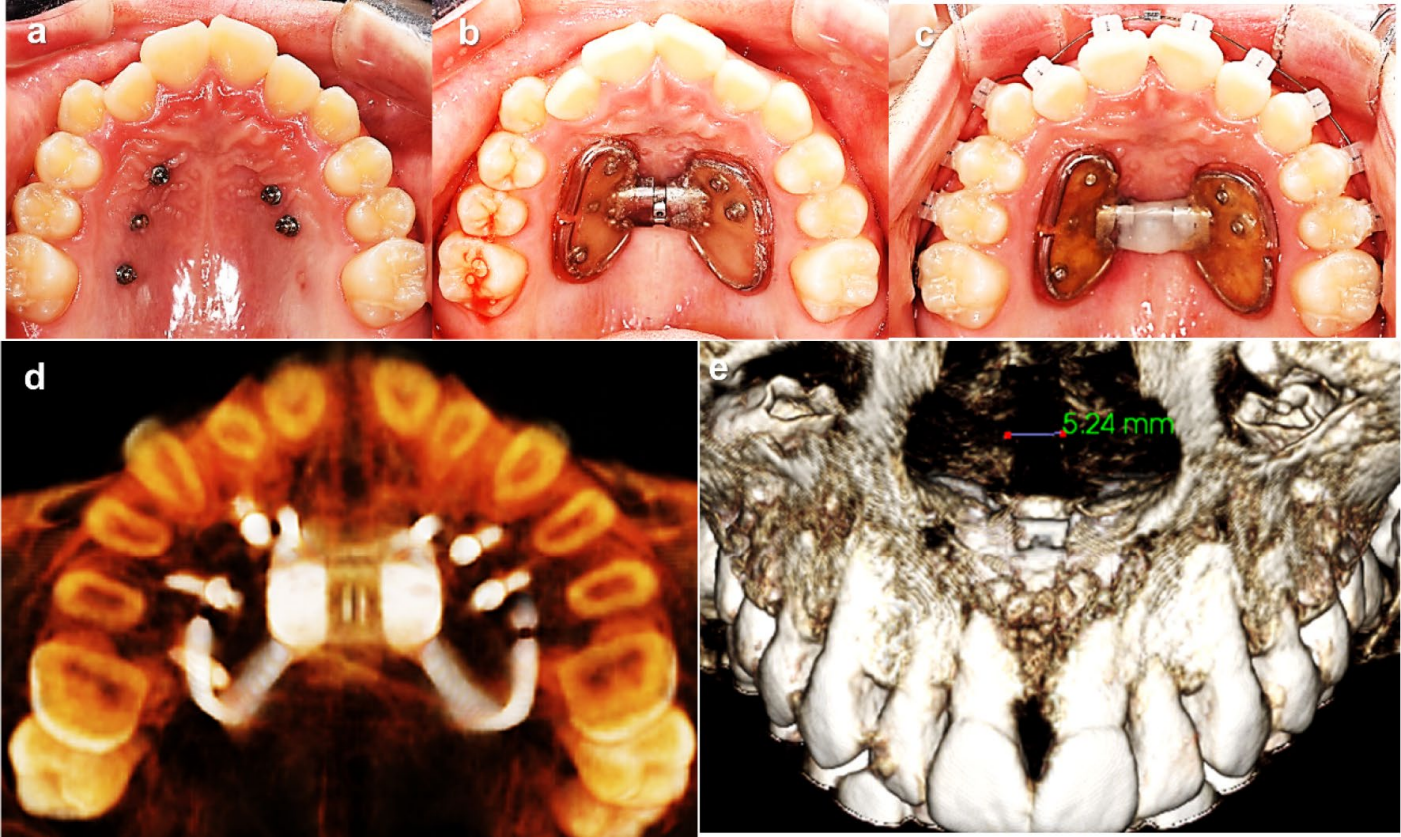
Clinical application of C-expander using self-drilling miniscrews. (**a**–**c**) Occlusal photographs of 13 years female patient. After placement of miniscrews (One miniscrew was loosened during healing period) (**b**), after C-expander application (**c**), after 7 mm of C-expander activation. (**d**, **e**) Cone beam computed tomography (CBCT) images of 17 years old female patient treated by six miniscrews assisted C-expander. 5.24 mm of suture separation was achieved after C-expander activation.

Orthodontic miniscrews were implanted on the palatal slope for the C-expander application by one orthodontist (S.H.K). The miniscrews were located between the roots of the canine and the first premolars, the first and second premolars, and the second premolar and first molars. The vertical insertion sites were basically determined at 7 mm below the cemento-enamel junction of each tooth. After marking the insertion site, the pre-drilling procedure was performed for the patients with type A miniscrew. The drill was positioned perpendicular at the palatal slope, and perforated just the cortical bone without changing the angle. The self-tapping miniscrew was then inserted passively through the drilled hole. Type B miniscrew, a self-drilling miniscrew, was also positioned perpendicular to the palatal slope but without pre-drilling procedure. It perforated cortical bone and inserted changing its angle gradually up to 30° to the palatal slope. After all the anchoring miniscrews were placed, the custom fabricated expander with an expansion screw was connected to the miniscrews using acrylic resin. The expansion screw of the C-expander was activated at a rate of two turns per week immediately after application until evidence of opening the midpalatal suture was confirmed by occlusal topography. After opening the suture, one turn per day was performed until the required expansion was achieved (Fig. 2)^41^.

### CBCT protocol

The CBCT images were acquired using the Alphard VEGA-3030 (Asahi Roentgen Co. Ltd., Kyoto, Japan); Field of view (FOV): 15.4 cm (diameter) × 15.4 cm (height), exposure time: 17 s, tube voltage: 80 kVp, tube current: 10 mA (adult), 3 mA (child). The data obtained were imported as DICOM-files using OnDemand-3D™ software (Cybermed, Daejeon, Korea) and ON3D software (3D ONS Inc, Seoul, Korea. http://www.3dons.net/en/).

### Measurements

**Figure 3:**
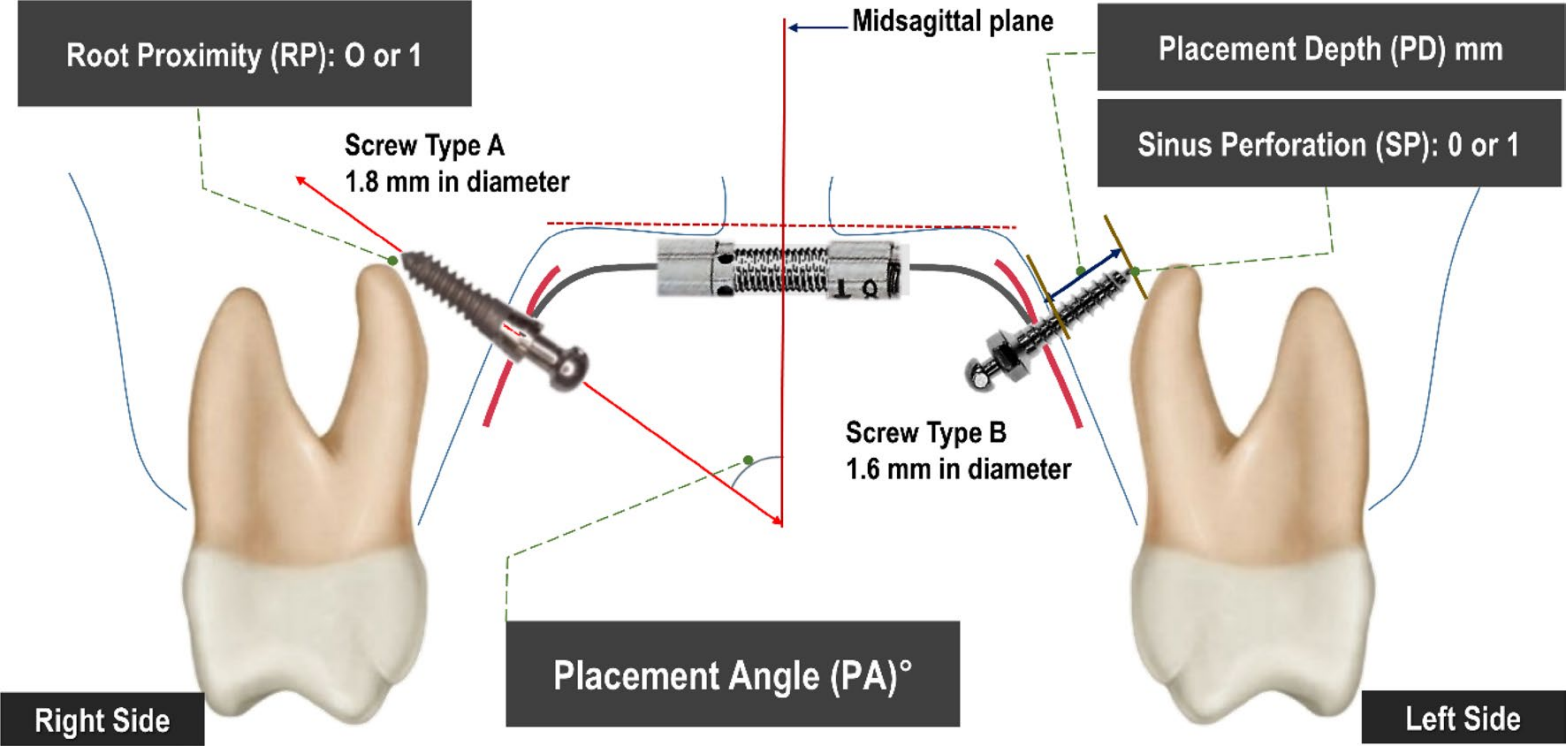
Schematic illustration of the measurements in this study. Screw Type A (C-implant; Cimplant Co, Seoul, Korea) was sand-blasted with a large-grit and acid etched (SLA) surface treated self-tapping miniscrew with a diameter of 1.8 mm and a length of 9.5 mm. Screw Type B (Bio-Action screw, Jin Biomed co., Bucheon, Korea) was a machined self-drilling miniscrew with a diameter of 1.6 mm and a length of 8.0 mm.

Two examiners performed all of the measurements on the CBCT images using the same computer and screen (resolution of 1920 × 1440 pixels) under ambient room lighting conditions. Placement depth (PD), placement angle (PA), root proximity (RP), and sinus perforation (SP) were evaluated (Fig. 3).

**Figure 4:**
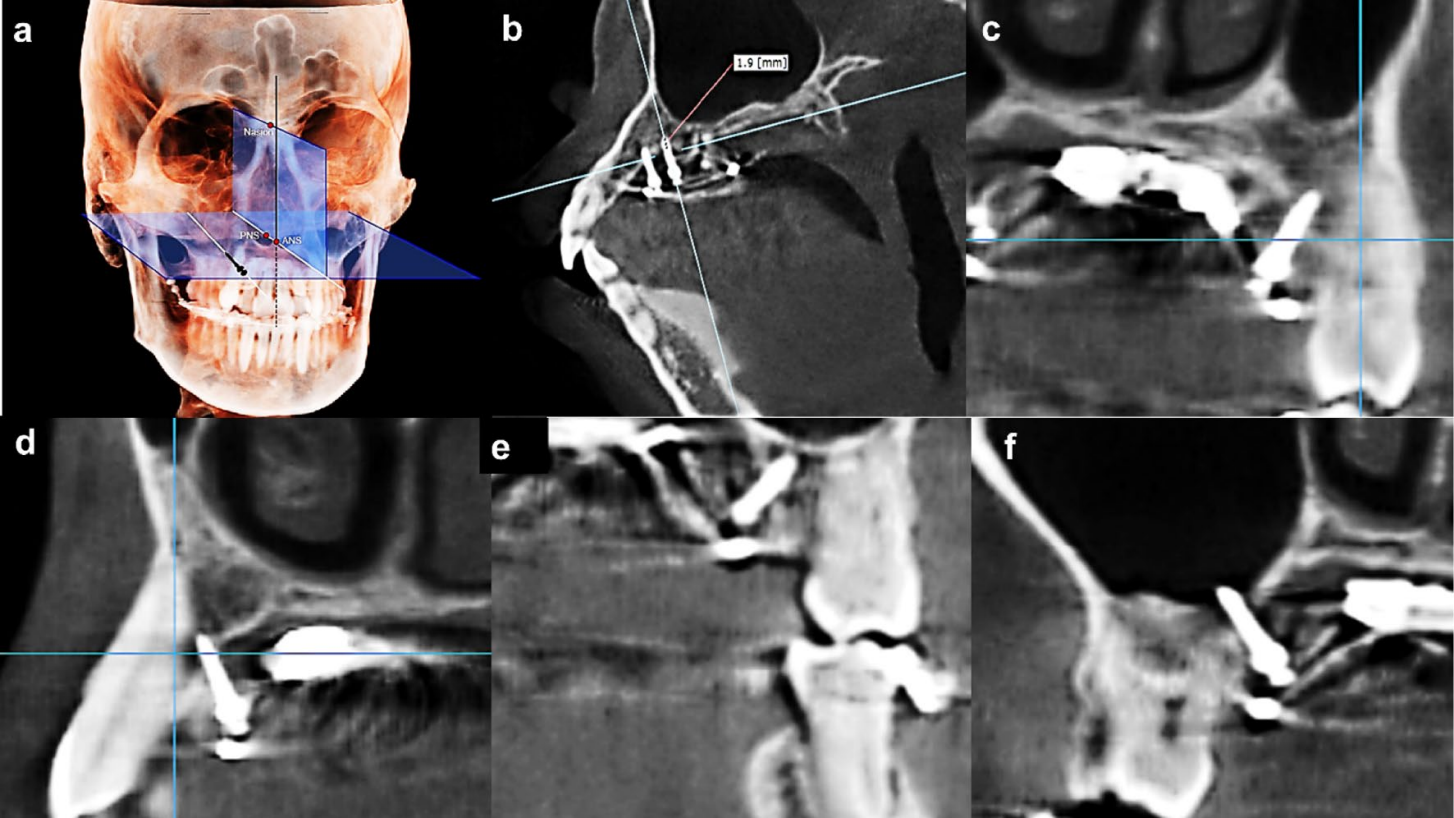
Miniscrew position on each plane. (**a**) Placement angle (PA) was measured using On3D. The plane through nasion (N), anterior nasal spine (ANS) and posterior nasal spine (PNS) was defined as the midsagittal plane, and PA was measured between the midsagittal plane and the straight line through the screw head and tip. (**b**) CBCT of adjusted sagittal view: placement depth (PD) of miniscrew around first premolar at right side, (**c**) CBCT image of frontal view: the surface of the miniscrew had no contact with left second premolar, (**d**) CBCT image of sagittal view: the tip of the miniscrew had contact with right canine, (**e**) CBCT image of frontal view: the tip of the miniscrew didn't perforate left maxillary sinus, (**f**) CBCT image of frontal view: the tip of the miniscrew perforated right maxillary sinus.

PD, RP, and SP were measured using OnDemand 3D software. PD was the actual length of the miniscrew that penetrated the bone. The PD could be measured with the adjusted sagittal image and cross-sectional image derived from the axial image (Fig. 4a).

Root proximity was defined as partial contact of the miniscrew surface with an adjacent root or periodontal ligament of the root in the adjusted sagittal images of the CBCT. Sinus perforation was defined as contact of the miniscrew with the sinus floor or the sinus membrane in the adjusted sagittal and cross-sectional image of CBCT (Fig. 4b). Root proximity and sinus perforation of the miniscrew were checked with the sagittal and frontal CBCT images. If there was conflict between observers about root proximity or sinus perforation, it was resolved with a follow-up discussion. Placement angle (PA) was measured using ON3D software with the automatic digitization of the landmarks function and the automatic re-orientation function that uses five landmarks: the nasion (N), left and right fronto-zygomatic points, right porion (Po), and right orbitale (Or). The first reference plane was the nasofronto-zygomatic plane. Then, the N point was registered as the origin (0, 0, 0) using Cartesian coordinate system. The 3D-coordinate values (x, y, z) of N, anterior nasal spine (ANS), and posterior nasal spine (PNS) were obtained from the patient's initial CBCT (before expansion) image. And those of the head and tip of the screw were obtained from the CBCT image taken after placing the miniscrews (Fig. 4c, d) ^42^. The plane through N, ANS and PNS was defined as the midsagittal plane, and PA was measured between the midsagittal plane and the straight line through the screw head and tip (Fig. 4e, f). These clinical variables were investigated: skeletal classification, screw type, placement sites and screw location.

### Statistical analysis

When the PD was 0 or the PA was less than 10°, it was regarded as a deviation of the miniscrew anchorage during the skeletal expansion process and excluded from the statistical analysis. To determine the inter-examiner reproducibility, the same examiners repeated the measurements on 20 sets of randomly selected CBCT data at 2 weeks’ intervals. The intraclass correlation coefficient (ICC: > 0.9) and inter-observer coefficient (ICC: 0.75–0.87) showed significant and excellent agreement. The Shapiro–Wilk normality test was used to examine normality of distribution of the outcomes measured. The Multivariable mixed linear model was used to compare root proximity, maxillary sinus perforation, placement depth and placement angle according to the skeletal classification, screw type, placement side and screw location. The Spearman correlation analysis was performed to confirm the correlation between placement depth and placement angle values. All statistical analyses were performed using SPSS version 22.0 software (SPSS Inc, Chicago, Ill).

## Supplementary Information

Supplementary Legends.

Supplementary Figure S1.

Supplementary Figure S2.

Supplementary Figure S3.

Supplementary Figure S4.

Supplementary Figure S5.
