# Supplementary material for: Geometry of anchoring miniscrew in the lateral palate that support a tissue bone borne maxillary expander affects neighboring root damage
Source: Sci Rep. 2021 Oct 6;11:19880. doi: 10.1038/s41598-021-99442-2 (PMC8494793; doi:10.1038/s41598-021-99442-2)
Supplement: Supplementary file 1 — Supplementary Legends. [file 41598_2021_99442_MOESM1_ESM.docx]

**Supplemental figure legends:**

**Supplemental Figure S1:** CBCT images of six miniscrews which showed deviated position. These data were excluded from the statistical analysis.

**Supplemental Figure S2:** CBCT images of 22 miniscrews which showed root proximity to maxillary canine.

**Supplemental Figure S3:** CBCT images of 15 miniscrews which showed root proximity to maxillary first premolar.

**Supplemental Figure S4:** CBCT images of 7 miniscrews which showed root proximity to maxillary second premolar.

**Supplemental Figure S5:** CBCT images of 14 miniscrews which showed root proximity to maxillary first molar.
