# Supplementary figures and images for: Geometry of anchoring miniscrew in the lateral palate that support a tissue bone borne maxillary expander affects neighboring root damage
Source: Sci Rep. 2021 Oct 6;11:19880. doi: 10.1038/s41598-021-99442-2 (PMC8494793; doi:10.1038/s41598-021-99442-2)

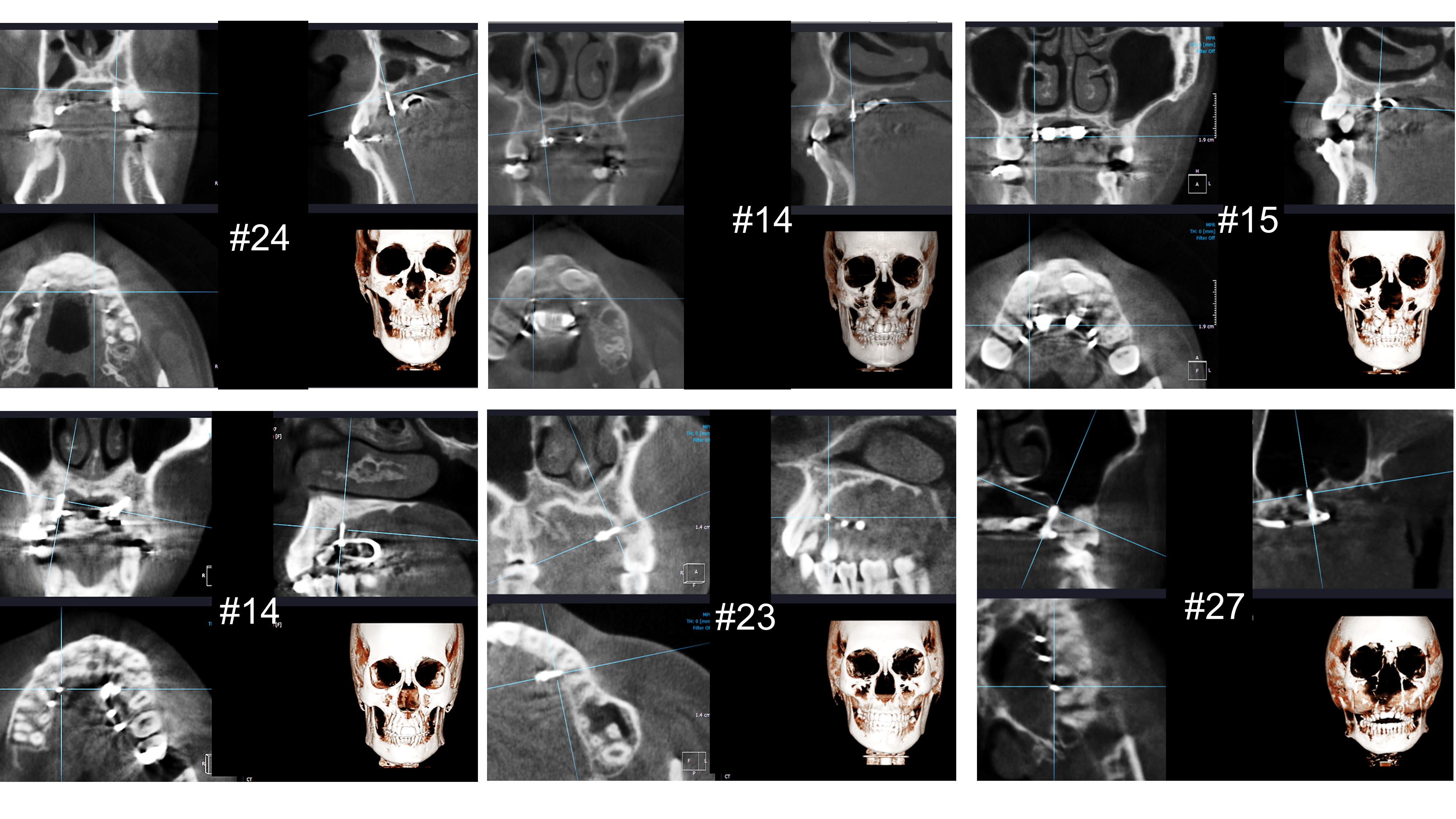

Supplement: Supplementary file 2 — Supplementary Figure S1. [file 41598_2021_99442_MOESM2_ESM.tif]

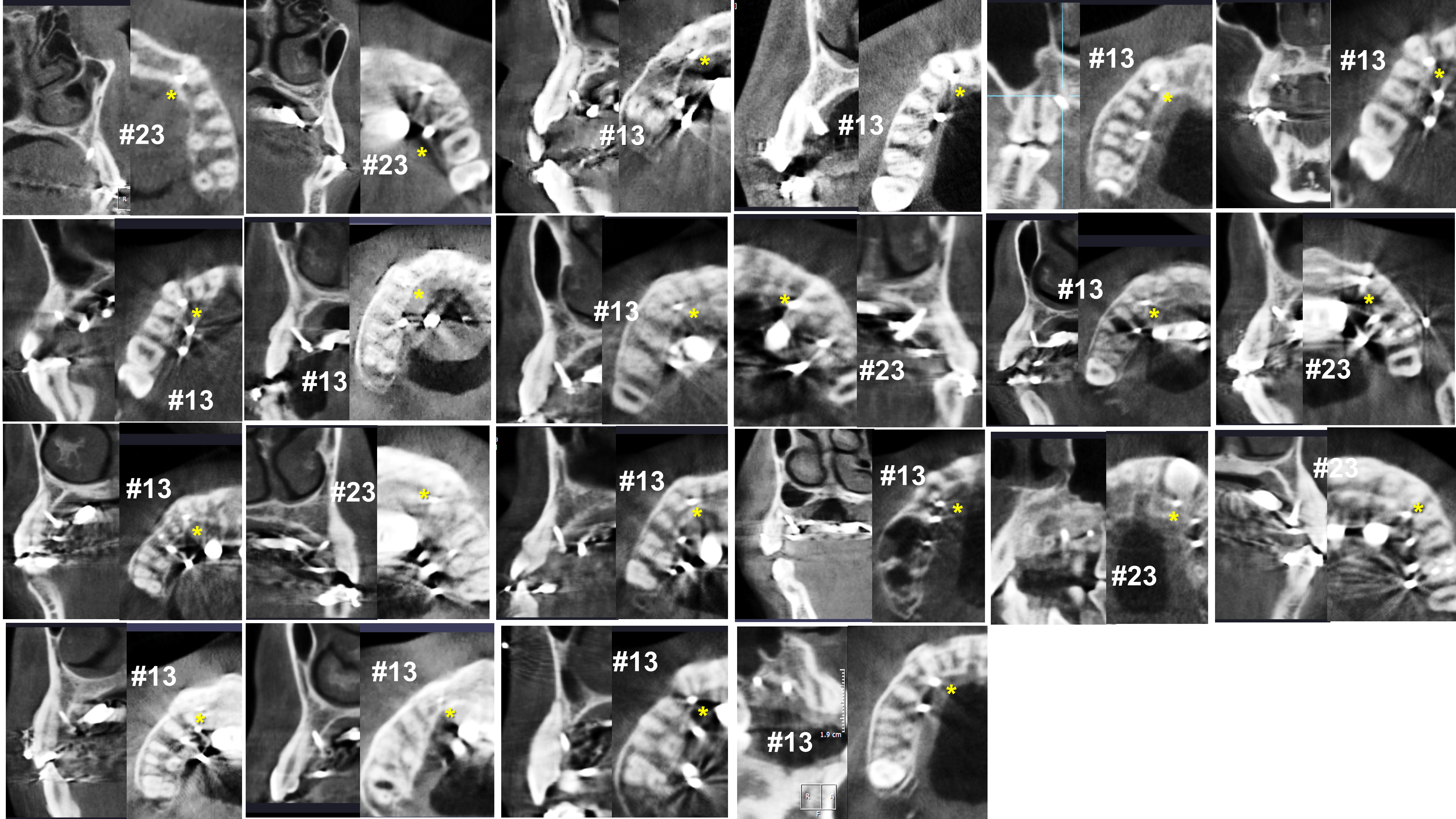

Supplement: Supplementary file 3 — Supplementary Figure S2. [file 41598_2021_99442_MOESM3_ESM.tif]

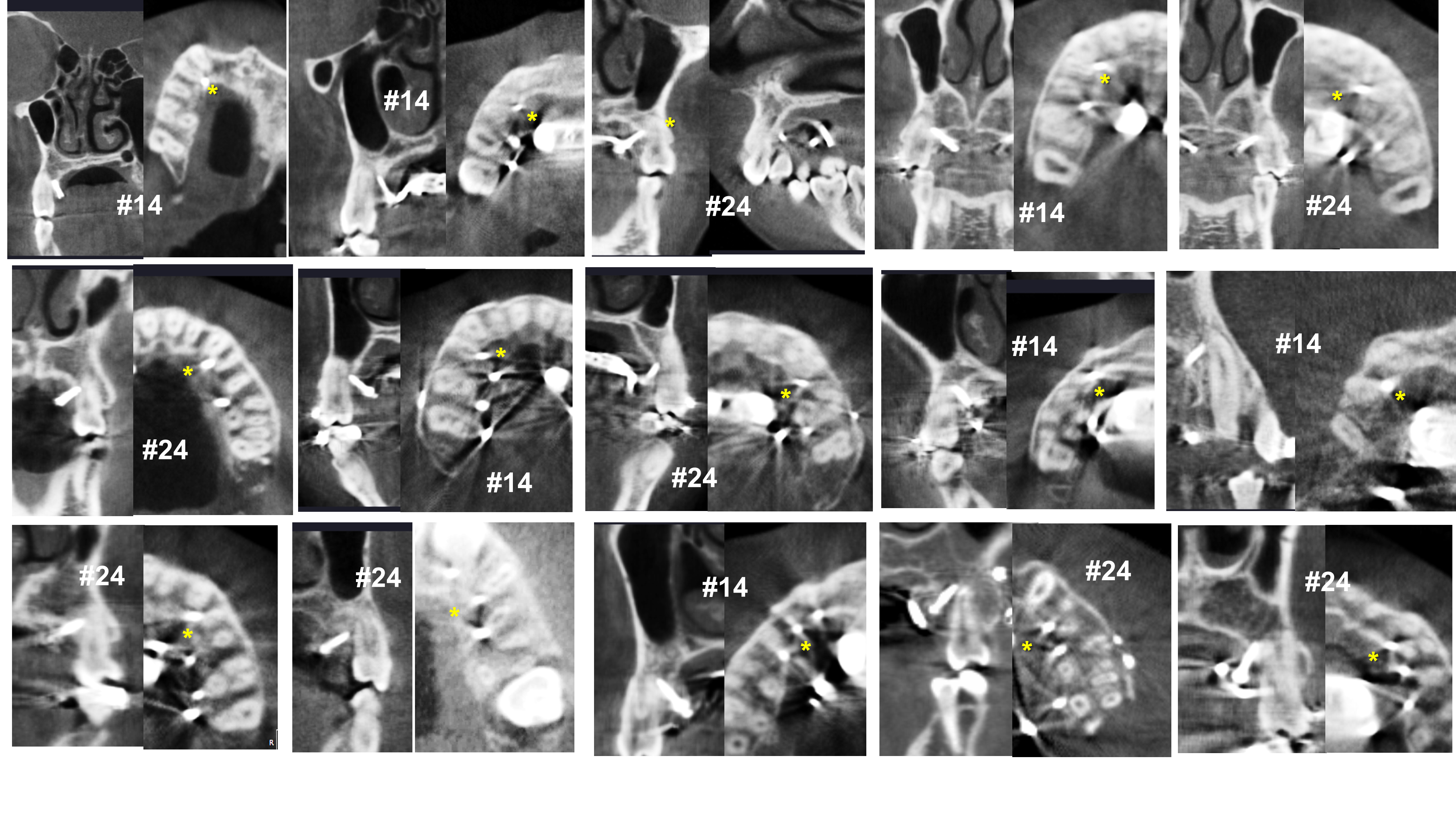

Supplement: Supplementary file 4 — Supplementary Figure S3. [file 41598_2021_99442_MOESM4_ESM.tif]

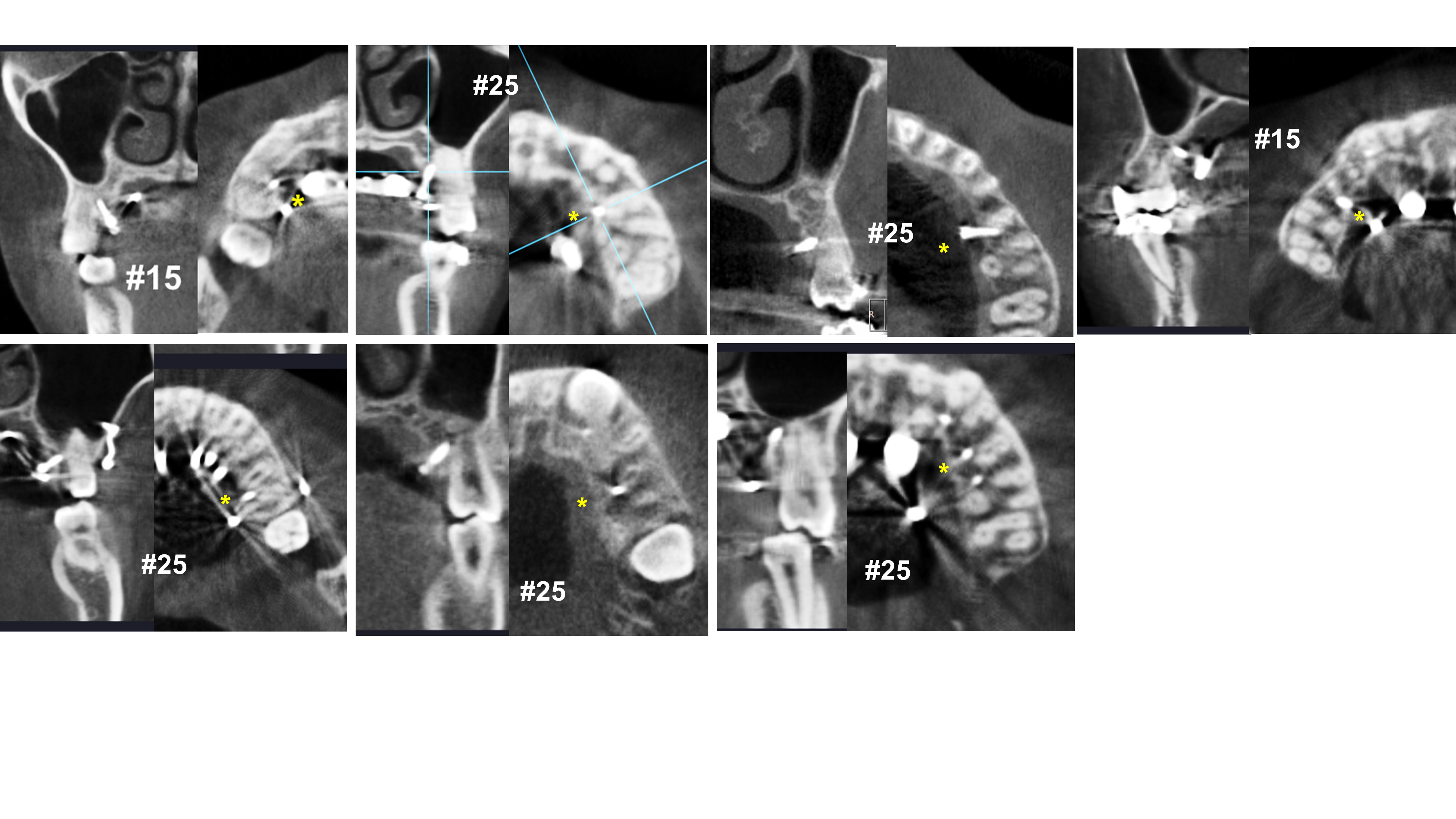

Supplement: Supplementary file 5 — Supplementary Figure S4. [file 41598_2021_99442_MOESM5_ESM.tif]

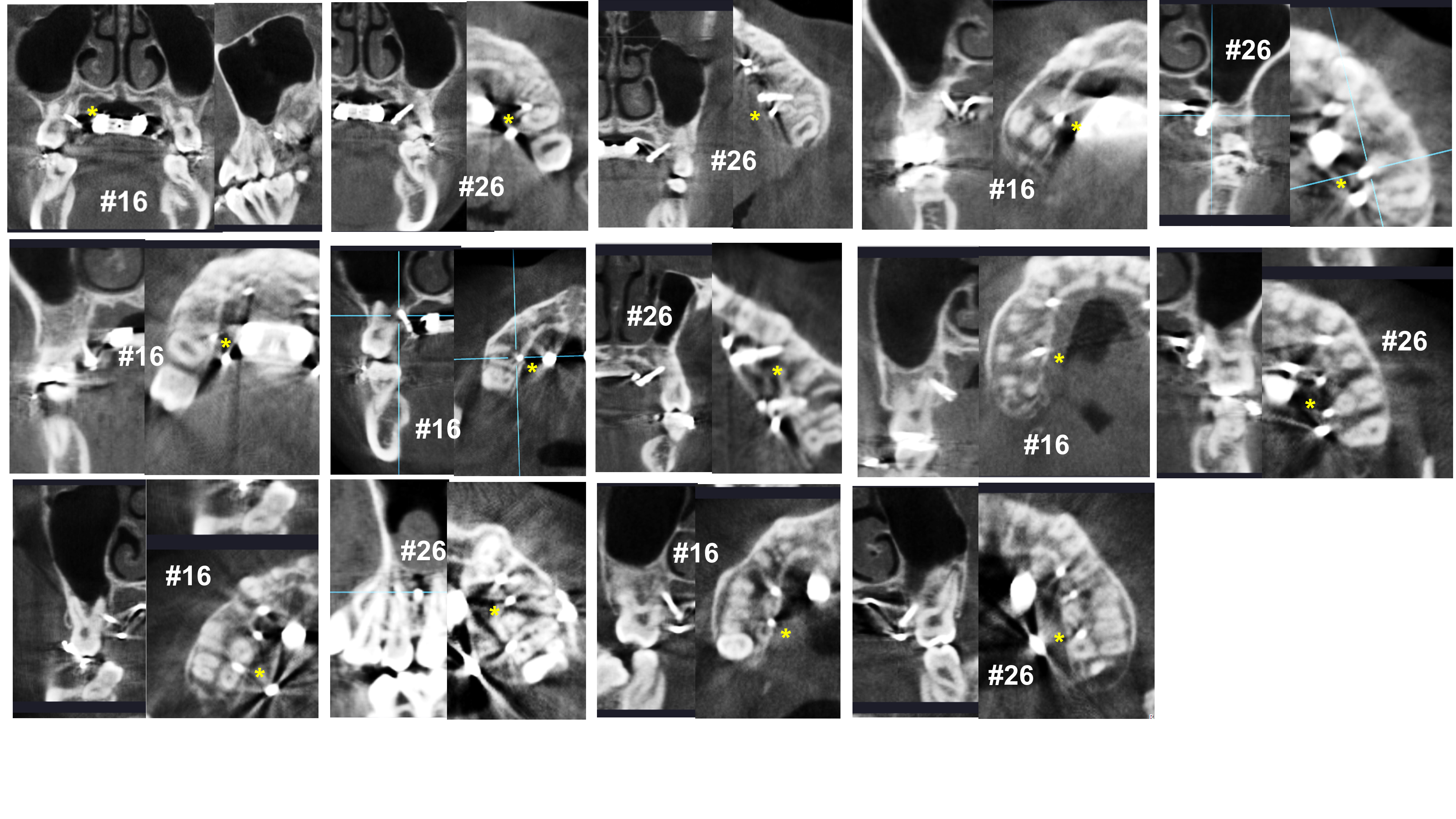

Supplement: Supplementary file 6 — Supplementary Figure S5. [file 41598_2021_99442_MOESM6_ESM.tif]
